# Dermoscopy, reflectance confocal microscopy, and high‐frequency ultrasound for the noninvasive diagnosis of morphea‐form basal cell carcinoma

**DOI:** 10.1111/srt.13197

**Published:** 2022-07-24

**Authors:** Li‐Wen Zhang, Xue Shen, Li‐Xin Fu, Hui‐Min Meng, Yong‐Hong Lu, Tao Chen, Rong‐Hua Xu

**Affiliations:** ^1^ Department of Dermatovenereology Chengdu Second People's Hospital Chengdu Sichuan China; ^2^ Institute of Dermatology Chengdu Second People's Hospital Chengdu Sichuan China

Dear Editor,

A 59‐year‐old female presented with a 5‐year history of a solitary skin‐colored nodule on the upper lip (Figure 1A). The lesion gradually enlarged without any discomfort. No skin inflammation or trauma was observed at the same location. She was in good health and stated no prior medical history. Dermoscopy manifested arborizing vessels with whitish structures presented on the pink background (Figure 1B). Reflectance confocal microscopy (RCM) showed numbers of bright tumor islands with peripheral palisading, cleft‐like dark spaces, and dilated canalicular vessels in the dermis (Figure 1C). High‐frequency ultrasound (HFUS) revealed an irregular hypoechoic dermal and hypodermal structure (Figure 1D). Histopathology revealed strands and nests of basaloid cells surrounding dense and sclerotic fibrous stroma in the dermis and subcutaneous tissue (Figure 1E). A diagnosis of morphea‐form basal cell carcinoma (BCC) was made. The patient was successfully treated with surgery, and follow‐up for 2 years showed no recurrence.

**FIGURE 1:**
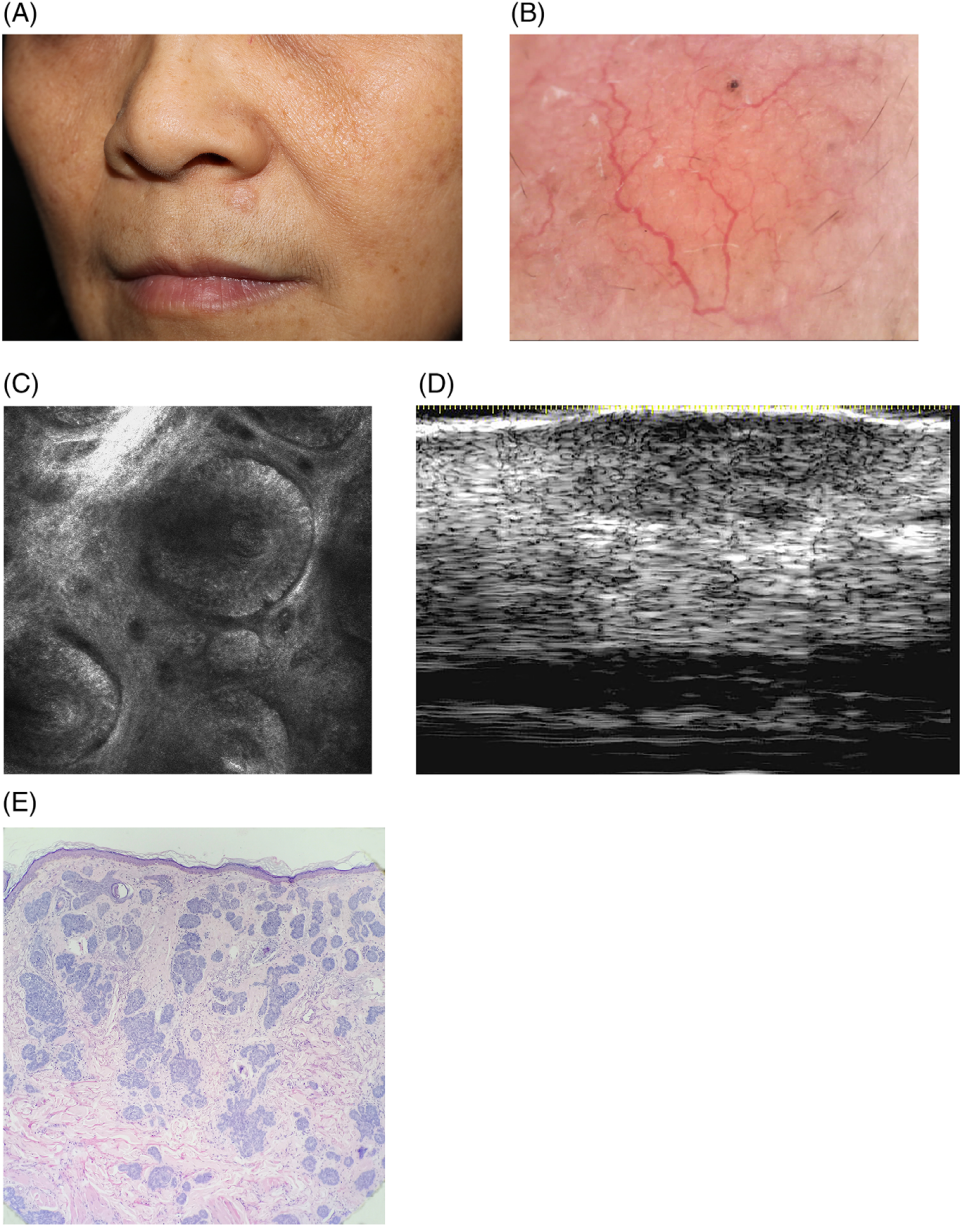
(A) A single skin‐colored nodule on the upper lip. (B) Dermoscopy: arborizing vessels with whitish structures on the pink background (×50 magnification). (C) RCM: bright tumor islands with peripheral palisading, cleft‐like dark spaces, and dilated canalicular vessels in the dermis (0.5 × 0.5 μm magnification). (D) Ultrasound: an irregular hypoechoic dermal and hypodermal structure (20 MHz) (E) Strands and nests of basaloid cells surrounding dense and sclerotic fibrous stroma in the dermis and subcutaneous tissue (HE ×100)

BCC is the most common cutaneous malignant neoplasm worldwide, and its incidence continues to increase. It is preferentially localized on photo‐exposed areas, particularly the face and the scalp. The most common subtype of BCC is nodular BCC, followed by superficial BCC. The more invasive and aggressive subtypes, such as metatypic, plexiform, and morphea‐form BCC, are rare and more challenging to diagnose on clinical grounds alone. As noninvasive and convenient imaging technologies, dermoscopy, RCM and HFUS are sensitive and specific tools for diagnosing BCC and can provide multimodal information for the management.

By visualizing cutaneous structures that are invisible to the naked eye, dermoscopy increases the accuracy of dermatologic examinations. The sensitivity and specificity of BCC diagnosis were significantly improved after adding dermoscopy to the naked‐eye examination.^1^ The sensitivity and specificity of dermoscopy for BCC diagnosis were 91.2% and 95%, respectively.^1^ Furthermore, the dermoscopic feature may aid in differentiating between BCC histopathologic subtypes.^2^ The main dermoscopic feature of morphea‐form BCC was pink‐white areas and/or fine arborizing vessels.^3^ However, dermoscopy can only observe the epidermal and superficial dermal structures. For deeper structural detection, RCM and HFUS are more advantageous.

RCM can supply image information closed to histologic resolution. Previous studies assessing RCM for BCC diagnosis reported varying sensitivity and specificity values ranging from 85%–97% and 89%–99%, respectively.^4^ Meanwhile, RCM and dermoscopy were helpful for preoperative defining lesional boundary and BCC histopathologic subtypes.^5^, ^6^, ^7^ On RCM, cords connected to the epidermis were seen in superficial BCC; big tumor islands, peritumoral collagen bundles and increased vascularization were relative to nodular BCC; and hyporefractile silhouettes associated with aggressive BCC.^6^ HFUS can provide reliable information about BCC, such as histopathological subtype differentiation, margin delineation, and tumor size assessment.^8^, ^9^ The recurrence risk of BCC can be assessed preliminary by quantifying the ultrasonographic examinations.^8^ The presence of seven or more hyperechogenic spots within the lesion has been associated with histological subtypes with a high risk of recurrence.^10^

A variety of imaging techniques with their own advantages can supply multimodal preoperative information for the management options that purpose to meet the needs of clinicians, except for improving the diagnostic accuracy of BCC.

This article has no funding source. The authors have no conflict of interest to declare. This content has not been published, nor has submitted for publication elsewhere. On behalf of all the contributors, I will act and guarantor and will correspond with the journal from this point onward. We hereby transfer, assign, or otherwise convey all copyright ownership, including any all rights incidental thereto, exclusively to the journal, if such work is published by the journal.

The patient in this manuscript has given written informed consent to the publication of their case details.

## References

[1] Reiter O , Mimouni I , Gdalevich M , et al. The diagnostic accuracy of dermoscopy for basal cell carcinoma: a systematic review and meta‐analysis. J Am Acad Dermatol. 2019;80(5):1380‐1388. 30582991 10.1016/j.jaad.2018.12.026

[2] Reiter O , Mimouni I , Dusza S , Halpern AC , Leshem YA , Marghoob AA . Dermoscopic features of basal cell carcinoma and its subtypes: a systematic review. J Am Acad Dermatol. 2021;85(3):653‐664. 31706938 10.1016/j.jaad.2019.11.008 PMC9366765

[3] Conforti C , Pizzichetta MA , Vichi S , et al. Sclerodermiform basal cell carcinomas vs. other histotypes: analysis of specific demographic, clinical and dermatoscopic features. J Eur Acad Dermatol Venereol. 2021;35(1):79‐87. 32401364 10.1111/jdv.16597

[4] Que SK , Grant‐Kels JM , Longo C , Pellacani G . Basics of confocal microscopy and the complexity of diagnosing skin tumors: new imaging tools in clinical practice, diagnostic workflows, cost‐estimate, and new trends. Dermatol Clin. 2016;34(4):367‐375. 27692444 10.1016/j.det.2016.05.001

[5] Lupu M , Voiculescu VM , Caruntu A , Tebeica T , Caruntu C . Preoperative evaluation through dermoscopy and reflectance confocal microscopy of the lateral excision margins for primary basal cell carcinoma. Diagnostics (Basel). 2021;11(1):120. 10.3390/diagnostics11010120. PMID: 33466602; PMCID: PMC7828674. 33466602 PMC7828674

[6] Lupu M , Popa IM , Voiculescu VM , et al. A retrospective study of the diagnostic accuracy of in vivo reflectance confocal microscopy for basal cell carcinoma diagnosis and subtyping. J Clin Med. 2019;8(4):449. 30987174 10.3390/jcm8040449 PMC6518285

[7] Longo C , Lallas A , Kyrgidis A , et al. Classifying distinct basal cell carcinoma subtype by means of dermatoscopy and reflectance confocal microscopy. J Am Acad Dermatol. 2014;71(4):716‐724. 24928707 10.1016/j.jaad.2014.04.067

[8] Halip IA , Vata D , Statescu L , et al. Assessment of basal cell carcinoma using dermoscopy and high frequency ultrasound examination. Diagnostics (Basel). 2022;12(3):735. 35328289 10.3390/diagnostics12030735 PMC8947530

[9] Bungardean RM , Serbanescu MS , Colosi HA , Crisan M . High‐frequency ultrasound: an essential non‐invasive tool for the pre‐therapeutic assessment of basal cell carcinoma. Rom J Morphol Embryol. 2021;62(2):545‐551. 35024743 10.47162/RJME.62.2.21 PMC8848273

[10] Wortsman X , Vergara P , Castro A , et al. Ultrasound as predictor of histologic subtypes linked to recurrence in basal cell carcinoma of the skin. J Eur Acad Dermatol Venereol. 2015;29(4):702‐707. 25200424 10.1111/jdv.12660

